# Sulfur supplementation enhances nitric oxide efficacy in reversal of chromium-inhibited Calvin cycle enzymes, photosynthetic activity, and carbohydrate metabolism in wheat

**DOI:** 10.1038/s41598-023-33885-7

**Published:** 2023-04-26

**Authors:** Mehar Fatma, Zebus Sehar, Noushina Iqbal, Ameena Fatima Alvi, Gholamreza Abdi, Charalampos Proestos, Nafees A. Khan

**Affiliations:** 1 Department of Botany, Aligarh Muslim University, Aligarh, 202002 India; 2 Department of Botany, School of Chemical and Life Sciences, Jamia Hamdard, New Delhi, 110062 India; 3 Department of Biotechnology, Persian Gulf Research Institute, Persian Gulf University, Bushehr, 75169 Iran; 4 Department of Chemistry, National and Kapodistrian University of Athens, Panepistimiopolis Zographou, 15771 Athens, Greece

**Keywords:** Plant development, Plant physiology, Plant signalling, Plant stress responses, Plant sciences

## Abstract

The present study demonstrated that exogenously-sourced nitric oxide (as SNP, sodium nitroprusside; NO donor) and sulfur (S) protected photosynthesis against chromium (Cr) stress in wheat (*Triticum* *aestivum* L. cv. HD 2851). Plants grown with 100 µM Cr exhibited higher reactive oxygen species (ROS) production, resulting in photosynthetic damage. The individual application of 50 µM NO increased carbohydrate metabolism as well as photosynthetic parameters, antioxidant system with higher transcriptional gene levels that encode the key enzymes for the Calvin cycle under Cr stress. These effects were more prominent when NO was applied with 1.0 mM SO_4_^2−^. An increase in the reduced glutathione (GSH) content obtained with NO was further enhanced by S and resulted in higher protection against Cr stress. The protective effect of NO with S against Cr toxicity on photosynthesis was reversed when buthionine sulfoximine (BSO; GSH biosynthetic inhibitor) was used. Application of BSO reversed the impact of NO plus S on photosynthesis under Cr stress, verifying that the ameliorating effect of NO was through S-assimilation and via GSH production. Thus, the availability of S to NO application can help reduce Cr toxicity and protect photosynthetic activity and expression of the Calvin cycle enzymes in leaves through the GSH involvement.

## Introduction

Heavy metal contamination has become a serious environmental hazard globally due to increasing concentrations beyond the permissible limit. Chromium (Cr) is the 17th most common element overall present in the earth’s mantle^1^. Although Cr is required at minimal levels by plants and animals, it is a serious environmental pollutant at higher concentrations. The anthropogenic activities along with natural sources result in the release of Cr in the air, water, and soil causing Cr pollution around the world^2^. Indeed, Cr rapidly enters the food chain and affects the well-being of all living things as a result of the pollution of agricultural fields and drinking water systems^3–5^. Due to Cr toxicity, plants exhibit delayed seed germination, damaged roots, reduced root growth, plant biomass, lower photosynthetic ability, lower crop yield, etc., and ultimately plant senescence. Moreover, it has been observed that Cr inhibits photosynthesis, and photosynthetic electron transport, along with the Calvin cycle enzymes deactivation^6^. Chromium has been found in various oxidation states, varying between 0 and + 6. Hexavalent Cr(VI) and trivalent Cr(III) are the two natural forms of Cr that are most stable. Because of its greater solubility and mobility in the water system, Cr(VI) is more hazardous than Cr(III)^1^. Plants take up both forms of Cr^7^. It is interesting to note that sulfate carriers actively transport Cr(VI) into plant cells^8^, and Cr(III), penetrates passively through the plant cell walls' cation exchange sites^9^. Furthermore, the carboxylic acids included in root exudates aid in the solubilization of Cr and uptake by plants^10^.

Numerous earlier studies have observed that phytohormones or mineral nutrients have a significant impact on plants' response to Cr toxicity in plants^11,12^. It was reported that the brinjal seedlings were protected from the damaging effects of Cr(VI) toxicity under high sulfur (S)^13^. Moreover, exogenous supplementation of signaling molecules such as nitric oxide (NO) and hydrogen sulfide (H_2_S) could boost plant efficiency by protecting key physiological processes such as photosynthesis by decreasing photo-inhibition, lipid peroxidation, stimulating the antioxidant defense system, and activating the transcription of stress-responsive genes under Cr stress^11,14^. Moreover, NO upregulates different genes involved in cell redox status to establish environmental stress tolerance^15^. In fact, for the upregulation of various cellular messengers, different S-derivatives activate signaling cascades such as NO, Ca^2+^, and abscisic acid^16–18^. It has been observed that NO helps to prevent oxidative damage via the production of reduced glutathione (GSH)^19^. On the other hand, S supplementation increased GSH content in plant tissues via increasing S absorption^20^. Moreover, NO also enhances the assimilation of S and interacts with specific molecules, reduces reactive oxygen species (ROS) damage, improves antioxidant defense, and protects the chloroplast of mustard^21^.

Nevertheless, the interactive outcome of NO and S on photosynthesis and growth, and the activity and expression of the Calvin cycle genes and carbohydrate metabolism under Cr stress are not available to date. A few studies on the role of exogenous NO and/or S in alleviating Cr toxicity have been reported^22–24^, but how exogenous NO application with S availability affects photosynthesis, the activity, and expression of the Calvin cycle enzymes, and carbohydrate metabolism in wheat need to be elucidated. This is important because environmental stress disrupts carbon metabolism by changing the expression and activity of carbon metabolism-related enzymes, the accumulation of starch, and the production of sucrose^25–27^. These disruptions are the fundamental cause of aberrant development and yield loss in plants under stress conditions. Moreover, wheat is the third most important cereal grain globally, possesses nutritional value and has been an increasing model system to study plant abiotic stress responses. It was reported that wheat is more vulnerable to Cr toxicity than the other crops, which resulted in lower plant growth, damaged roots, etc., and ultimately, reduced grain yield^28^. A detailed study on photosynthesis including the efficiency of the Calvin cycle enzymes, gas exchange parameters, and carbohydrate metabolism in response to S and NO supplementation under Cr stress has not been done so far. Thus, the reported research was carried out in order to test the contribution of S in enhancing the efficacy of exogenously-sourced NO in the protection of photosynthetic characteristics and the Calvin cycle enzymes expression as well as to explore its relationship with an accumulation of carbohydrates for adaptation of wheat plants grown in Cr stress.

## Results

### Supplementation of NO with S enhanced the enzymatic antioxidant system under Cr stress

In order to verify if NO and/or S could potentially reduce oxidative stress, we evaluated their effects on the content of hydrogen peroxide (H_2_O_2_) and thiobarbituric acid reactive substances (TBARS) (Table 1). We observed that wheat plants exposed to Cr stress exhibited considerably increased oxidative stress, which was associated with elevated H_2_O_2_ (+ 113.2%) and TBARS (+ 107.6%) content levels in comparison to the control. However, the treatment of S reduced oxidative stress by decreasing the levels of H_2_O_2_ and TBARS by 49.3% and 43.5%, respectively. Also, NO treatment decreased the content of H_2_O_2_ by 42.7% and TBARS by 28.2% in comparison with the control. Interestingly, NO treatment with S maximally reduced the levels of H_2_O_2_ and TBARS under Cr stress in comparison with the control and Cr-treated wheat plants. However, in the absence of stress, the individual applications of S or NO increased the antioxidant enzyme activity of ascorbate peroxidase (APX) by (95.5 and 76.7%), glutathione reductase (GR) by (60.1 and 53.0%), and catalase (CAT) by (70.5 and 61.3%), respectively, compared with the control plants. The antioxidant enzyme activity was increased to its highest level when S and NO were applied together, with an increase in the activity of APX (161.6 and 73.3%), GR (60.1% and 53.0%), and CAT (84.3% and 56.4%) in comparison with the control and Cr-stressed plants, respectively. Likewise, in the presence of Cr-stress, treatment of S with NO stimulated the maximal increase in the activity of APX, GR, and CAT compared to the control and Cr-treated plants.

**Table 1:** H_2_O_2_ and TBARS content, and activity of CAT, APX, and GR in wheat (*Triticum* *aestivum* L.) leaves at 30 DAS.

Treatments	H_2_O_2_ content (nmol g^−1^ DW)	TBARS content (nmol g^−1^ DW)	CAT activity (U mg^−1^ protein min^−1^)	APX activity (U mg^−1^ protein min^−1^)	GR activity
Control	15.2 ± 0.89	03.9 ± 0.22	121 ± 3.8	1.22 ± 0.057	0.204 ± 0.007
Cr	32.4 ± 0.98***	08.1 ± 0.23***	140 ± 4.0*	1.69 ± 0.063**	0.248 ± 0.009*
S	07.7 ± 0.76*	02.2 ± 0.18*	203 ± 5.4**	2.19 ± 0.096***	0.317 ± 0.013**
NO	08.7 ± 0.66*	02.8 ± 0.19*	192 ± 5.2**	1.98 ± 0.091**	0.303 ± 0.012**
NO + S	03.5 ± 0.54**	1.39 ± 0.14***	219 ± 5.8***	2.93 ± 0.112***	0.365 ± 0.014***
S + Cr	11.2 ± 0.56*	3.09 ± 0.19*	177 ± 4.4**	1.80 ± 0.085**	0.291 ± 0.011**
NO + Cr	12.8 ± 0.76*	3.27 ± 0.20*	162 ± 4.1**	1.73 ± 0.076**	0.263 ± 0.010**
NO + S + Cr	05.3 ± 0.65**	1.81 ± 0.17***	204 ± 5.6**	2.69 ± 0.108***	0.340 ± 0.012***

Plants were supplemented with 50 µM NO and/or treated with S (1.0 mM SO_4_^2−^) in the presence or absence of 100 µM Cr. The data are displayed as mean ± SE (*n* = 4). As established by Tukey’s posthoc multiple comparison test, values that differ significantly between the control and treatments are denoted with an asterisk (*p < 0.05, **p < 0.01, ***p < 0.001). *APX* ascorbate peroxidase, *CAT* catalase, *Cr* chromium, *DAS* d after sowing, *DW* dry weight, *GR* glutathione reductase, *H*_*2*_*O*_*2*_ hydrogen peroxide, *NO* nitric oxide, *S* sulfur, *TBARS* thiobarbituric acid reactive substances.

### Application of NO and S stimulated S-assimilation under Cr stress

**Figure 1:**
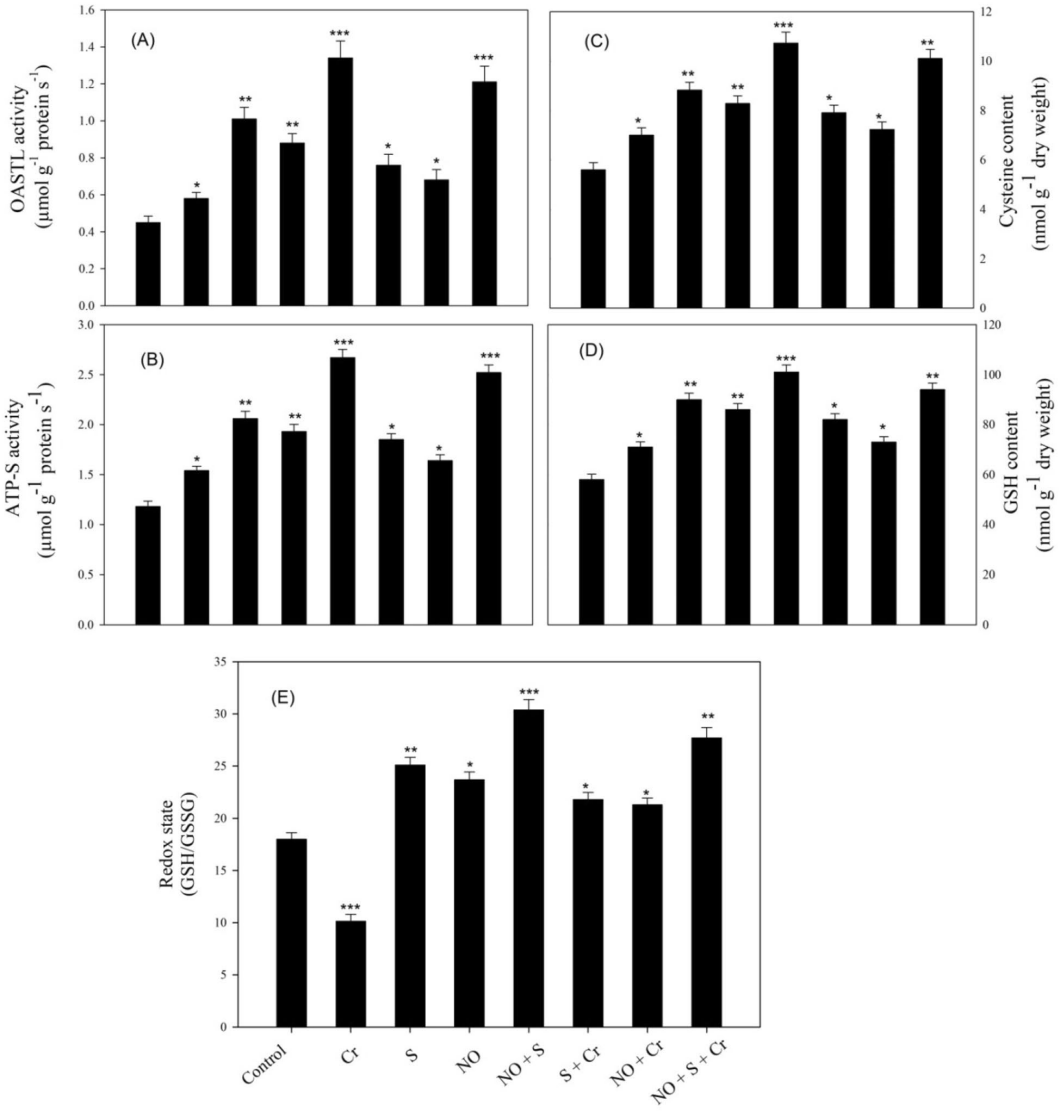
The activity of OASTL (**A**), ATP-S (**B**), the content of Cys (**C**), GSH (**D**), and redox state (**E**) in wheat (*Triticum* *aestivum* L.) leaves at 30 DAS. Plants were supplemented with 50 µM NO and/or treated with S (1.0 mM SO_4_^2−^) in the presence and absence of 100 µM Cr. The data is displayed as mean ± SE (*n* = 4). As established by Tukey’s posthoc multiple comparison test, values that differ significantly between the control and treatments are denoted with an asterisk (*p < 0.05, **p < 0.01, ***p < 0.001). *ATP-S* ATP-sulfurylase, *Cys* cysteine, *Cr* chromium, *DAS* d after sowing, *GSH* glutathione, *NO* nitric oxide, *OASTL* O-acetylserine (thiol) lyase, *S* sulfur.

The present study found that Cr stress stimulated the activity of ATP-sulfurylase (ATP-S) and *O*-acetylserine(thiol)lyase (OASTL) enzymes, which are the key enzymes of S-assimilation (Fig. 1). The activity of these enzymes was further enhanced by the simultaneous supply of NO and S compared to the control plants. Compared to the control and Cr stressed plants, ATP-S activity was maximally increased by (113.5% and 63.6%), respectively, with the supply of NO and S together. The activity of OASTL was enhanced by about 2.68 and 2 times in comparison to the control and Cr-treated plants, respectively. Moreover, Cr-stress increased the content of cysteine (Cys), GSH, and redox state in comparison to the control, which was substantially increased by the application of S or NO in comparison to the control under no stress. However, the addition of NO plus S to plants grown with Cr or under no stress conditions resulted in a notable rise in the content of Cys and GSH by (80.3% and 91.6%), and (70.9% and 83.6%), respectively, in comparison to the control. Similarly, the redox state increased by 53.8% and 68.8% with NO and S with Cr stress and no stress, respectively, when it was compared with the control plants.

### Application of NO with S improved photosynthetic efficiency and growth under Cr stress

To investigate the effects of S with NO on the alleviation of Cr-stress inhibited gas exchange and growth attributes, plants were grown with or without Cr (Table 2). The study showed that the Cr stress decreased net photosynthesis (*Pn*), stomatal conductance (*Gs*), and intercellular CO_2_ concentration (*Ci*) by 37.6%, 23.5%, and 32.0%, respectively, and the chlorophyll (Chl) content value by 42.7% compared to the control plants. Likewise, compared to the control, Cr-stress decreased leaf area, plant fresh weight, and plant dry weight by 44.4%, 62.3%, and 48.3%, respectively. But, the individual NO or S treatment under no-stress enhanced the photosynthetic and growth parameters. However, the highest increase was noted with their combined application, where *Pn*, *Gs,* *Ci*, and Chl content increased by 88.8%, 58.3%, 68.8%, and 74.2%, respectively, compared to the control. Similarly, NO plus S under the Cr stress significantly declined the negative impact of Cr toxicity on photosynthetic efficiency by increasing gas exchange attributes and Chl content in comparison with the control and Cr stress. Plants treated exogenously with NO or S, individually or in combined form, performed better than plants under Cr stress and exhibited considerably higher values for leaf area and plant dry weight. However, leaf area maximally increased by 61.6%, plant fresh weight by 34.1%, and plant dry weight increased by 72.5%, respectively, under Cr stress treated simultaneously with NO and S in comparison with the control.

**Table 2:** Gas exchange parameters of *Pn*, *Gs*, and *Ci*, and Chl content, and plant fresh and dry weight of wheat (*Triticum* *aestivum* L.) leaves at 30 DAS. Plants were supplemented with 50 µM NO and/or treated with S (1.0 mM SO_4_^2−^) in the presence and absence of 100 µM Cr.

Treatments	Pn (µmol CO_2_ m^−2^ s^−1^)	Gs (mmol m^−2^ s^−1^)	Ci (µmol CO_2_ mol^−1^)	Chl content (SPAD value)	Leaf area (cm^2^ plant^−1^)	Plant fresh weight (g plant^−1^)	Plant dry weight (g plant^−1^)
Control	11.6 ± 0.61	370 ± 11.1	250 ± 5.9	20.6 ± 1.18	107.0 ± 3.9	3.90 ± 0.14	1.31 ± 0.060
Cr	07.3 ± 0.55*	283 ± 9.30*	170 ± 4.5*	11.8 ± 1.11**	059.4 ± 2.5**	1.47 ± 0.04**	0.67 ± 0.054**
S	18.9 ± 0.75**	481 ± 18.7*	359 ± 6.3*	30.3 ± 1.62**	160.7 ± 5.3**	4.88 ± 0.12***	02.2 ± 0.087***
NO	18.3 ± 0.71**	474 ± 18.5*	338 ± 6.1*	27.4 ± 1.60*	149.4 ± 5.1*	4.66 ± 0.11**	1.96 ± 0.083**
NO + S	22.1 ± 0.88***	586 ± 22.3**	422 ± 6.8**	35.9 ± 1.85***	190.0 ± 5.8***	5.96 ± 0.19***	2.43 ± 0.094***
S + Cr	15.7 ± 0.68*	443 ± 17.0*	319 ± 5.9*	25.1 ± 1.55*	140.1 ± 4.8*	4.43 ± 0.11*	01.6 ± 0.075*
NO + Cr	13.5 ± 0.67*	401 ± 15.1ns	300 ± 4.9*	23.7 ± 1.45*	128.0 ± 4.7*	4.18 ± 0.09*	1.49 ± 0.067*
NO + S + Cr	20.6 ± 0.81***	536 ± 20.8**	377 ± 6.5**	33.5 ± 1.70***	173.0 ± 5.5***	5.23 ± 0.17***	2.26 ± 0.090***

The data is displayed as mean ± SE (*n* = 4). As established by Tukey’s posthoc multiple comparison test, values that differ significantly between the control and treatments are denoted with an asterisk (*p < 0.05, **p < 0.01, ***p < 0.001). *Chl* chlorophyll, *Ci* intercellular CO_2_ concentration, *DAS* d after sowing, *Cr* chromium, *Gs* stomatal conductance The data is displayed as mean ± SE (*n* = 4). As established by Tukey’s posthoc multiple comparison test, values that differ significantly between the control and treatments are denoted with an asterisk (*p < 0.05, **p < 0.01, ***p < 0.001). *Chl* chlorophyll, *Ci* intercellular CO_2_ concentration, *DAS* d after sowing, *Cr* chromium, *Gs* stomatal conductance, *NO* nitric oxide, *ns* non-significant, *Pn* net photosynthesis, *S* sulfur, *NO* nitric oxide, *ns* non-significant, *Pn* net photosynthesis, *S* sulfur.

**Figure 2:**
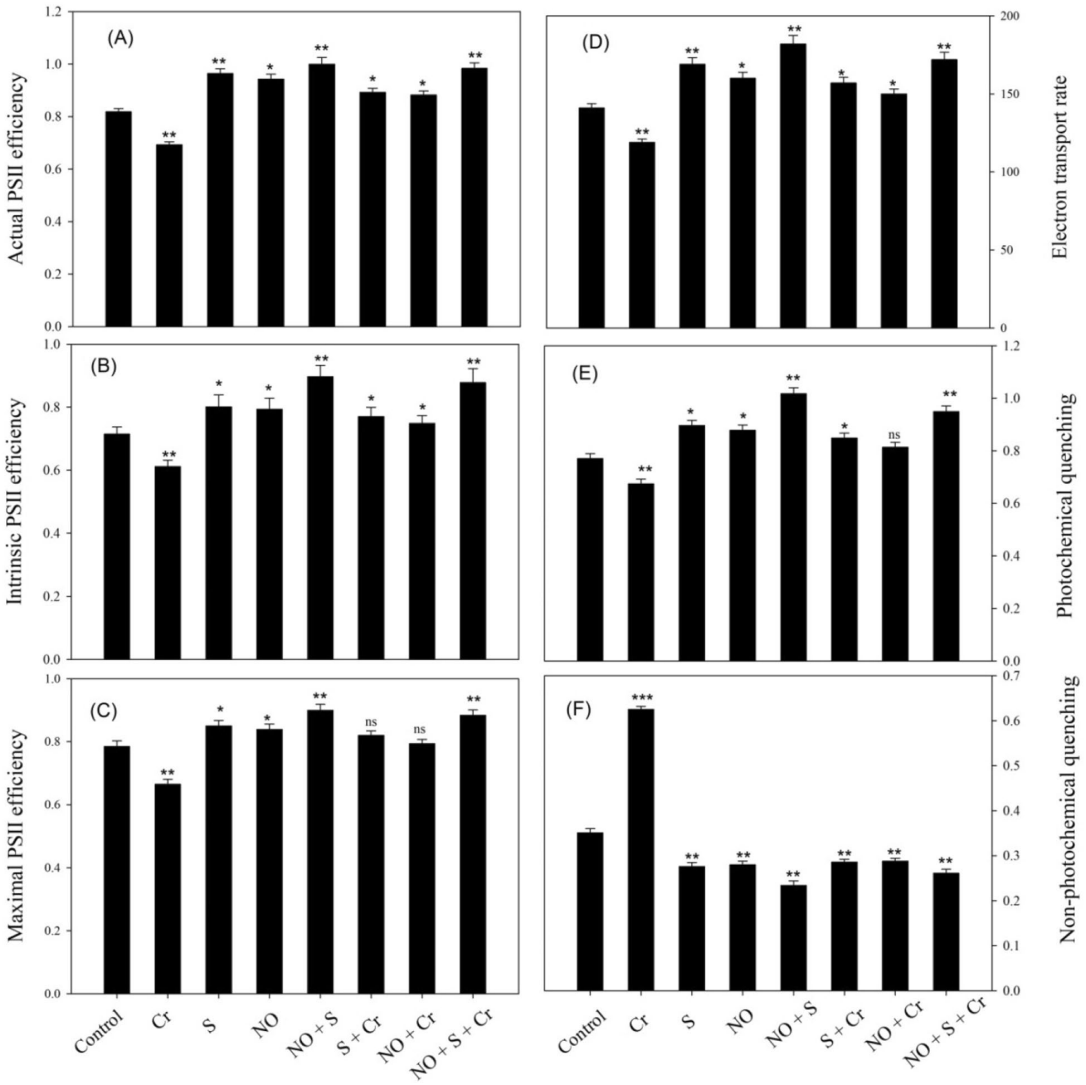
Actual PS II efficiency (**A**), intrinsic PS II efficiency (**B**), maximal PS II efficiency (**C**), electron transport rate (**D**), photochemical quenching (**E**), and non-photochemical efficiency (**F**) in wheat (*Triticum* *aestivum* L.) leaves at 30 DAS. Plants were supplemented with 50 µM NO and/or treated with S (1.0 mM SO_4_^2−^) in the presence and absence of 100 µM Cr. The data is displayed as mean ± SE (*n* = 4). As established by Tukey’s posthoc multiple comparison test, values that differ significantly between the control and treatments are denoted with an asterisk (*p < 0.05, **p < 0.01, ***p < 0.001). *Cr* chromium, *DAS* d after sowing, *NO* nitric oxide, *S* sulfur.

Similarly, plants grown under Cr stress showed decreased actual PS II efficiency, maximum PS II efficiency, intrinsic PS II efficiency, photochemical quenching (qP), and electron transport rate (ETR) by 15.3%, 13.7%, 14.4%, 12.4%, and 15.6%, respectively, in comparison to the control; nevertheless, non-photochemical quenching (NPQ) increased by 78.0% (Fig. 2). Therefore, a significant increase in actual PS II efficiency by (20.2% and 29.0%), maximal PS II efficiency by (14.6% and 32.9%), intrinsic PS II efficiency by (22.8% and 43.4%), qP by (23.2% and 40.8%), ETR by (21.9% and 44.5%), and a decreased in NPQ by (25.6% and 58.2%) was acquired with NO plus S under Cr-stress, respectively, compared with the control and Cr-stress plants.

### Application of NO with S increases the activity and gene expression of the Calvin cycle enzymes and carbohydrate metabolism

Plants subjected to Cr stress showed significant reductions in the activity of Rubisco, fructose-1,6-bisphosphatase aldolase (FBP aldolase), fructose-1,6-bisphosphatase (FBPase), and sedoheptulose-1,7-bisphosphatase (SBPase) by 42.8%, 38.4%, 37.3%, and 31.1%, respectively, in comparison to the control (Table 3). The individual application of NO or S enhanced the activities of Rubisco, FBPase, FBP aldolase, and SBPase, but a maximal increase was noted with the combined application of S and NO. A significant increase in the Rubisco activity by 54.7% and 52.3, FBP aldolase by 38.4% and 30.7%, FBPase by 46.1% and 43.9%, and SBPase by 44.1% and 32.4% were obtained with both NO and S in comparison to control under no stress and Cr-treated plants, respectively.

**Table 3:** The activity of Rubisco, FBPase, FBP aldolase, SBPase, and the content of non-structural carbohydrate (NSC), starch, and soluble sugars in wheat (*Triticum* *aestivum* L.) leaves at 30 DAS.

Treatments	Rubisco activity (µmol CO_2_ mg^−1^ protein min^−1^)	FBPase activity (µmol NADPH mg^−1^ protein min^−1^)	FBP aldolase activity (µmol NADH mg^−1^ protein min^−1^)	SBPase activity (µmol mg^−1^ protein s^−1^)	Total NSC content (mg g^−1^ dry weight)	Starch content (mg g^−1^ dry weight)	Soluble sugars content (mg g^−1^ dry weight)
Control	0.84 ± 0.020	0.91 ± 0.026	0.52 ± 0.020	0.77 ± 0.023	54.1 ± 4.1	23.6 ± 1.3	30.5 ± 2.6
Cr	0.48 ± 0.017**	0.57 ± 0.020**	0.32 ± 0.010**	0.53 ± 0.017*	40.9 ± 3.2*	18.1 ± 1.2*	22.8 ± 2.2*
S	1.25 ± 0.026**	1.28 ± 0.041**	0.66 ± 0.025*	0.98 ± 0.028*	66.8 ± 4.5*	29.2 ± .9*	37.6 ± 3.4*
NO	1.22 ± 0.025**	1.25 ± 0.032**	0.63 ± 0.015*	0.94 ± 0.025*	65.4 ± 4.8*	28.5 ± 1.5*	36.9 ± 3.2*
NO + S	1.30 ± 0.035**	1.33 ± 0.058***	0.72 ± 0.030**	1.11 ± 0.032**	74.9 ± 7.5**	32.1 ± 2.4**	42.8 ± 3.8**
S + Cr	1.19 ± 0.029*	1.21 ± 0.030*	0.61 ± 0.015*	0.89 ± 0.022*	61.0 ± 6.2*	27.2 ± 0.4*	35.0 ± 3.0*
NO + Cr	1.15 ± 0.028*	1.17 ± 0.029*	0.57 ± 0.014 ns	0.81 ± 0.020 ns	55.0 ± 4.2 ns	26.7 ± 0.3*	33.7 ± 2.8*
NO + S + Cr	1.28 ± 0.030**	1.31 ± 0.037***	0.68 ± 0.028**	1.02 ± 0.030*	71.2 ± 5.8*	30.5 ± 2.2*	40.7 ± 3.5**

Plants were supplemented with 50 µM NO and/or treated with S (1.0 mM SO_4_^2−^) in the presence and absence of 100 µM Cr. The data is displayed as mean ± SE (*n* = 4). As established by Tukey’s posthoc multiple comparison test, values that differ significantly between the control and treatments are denoted with an asterisk (*p < 0.05, **p < 0.01, ***p < 0.001). *Cr* chromium, *DAS* d after sowing; fructose-1,6-bisphosphatase, *FBPase* FBP aldolase, fructose-1,6-bisphosphatase aldolase, *NO* nitric oxide, *ns* non-significant, *S* sulfur, *SBPase* sedoheptulose-1,7-bisphosphatase.

**Figure 3:**
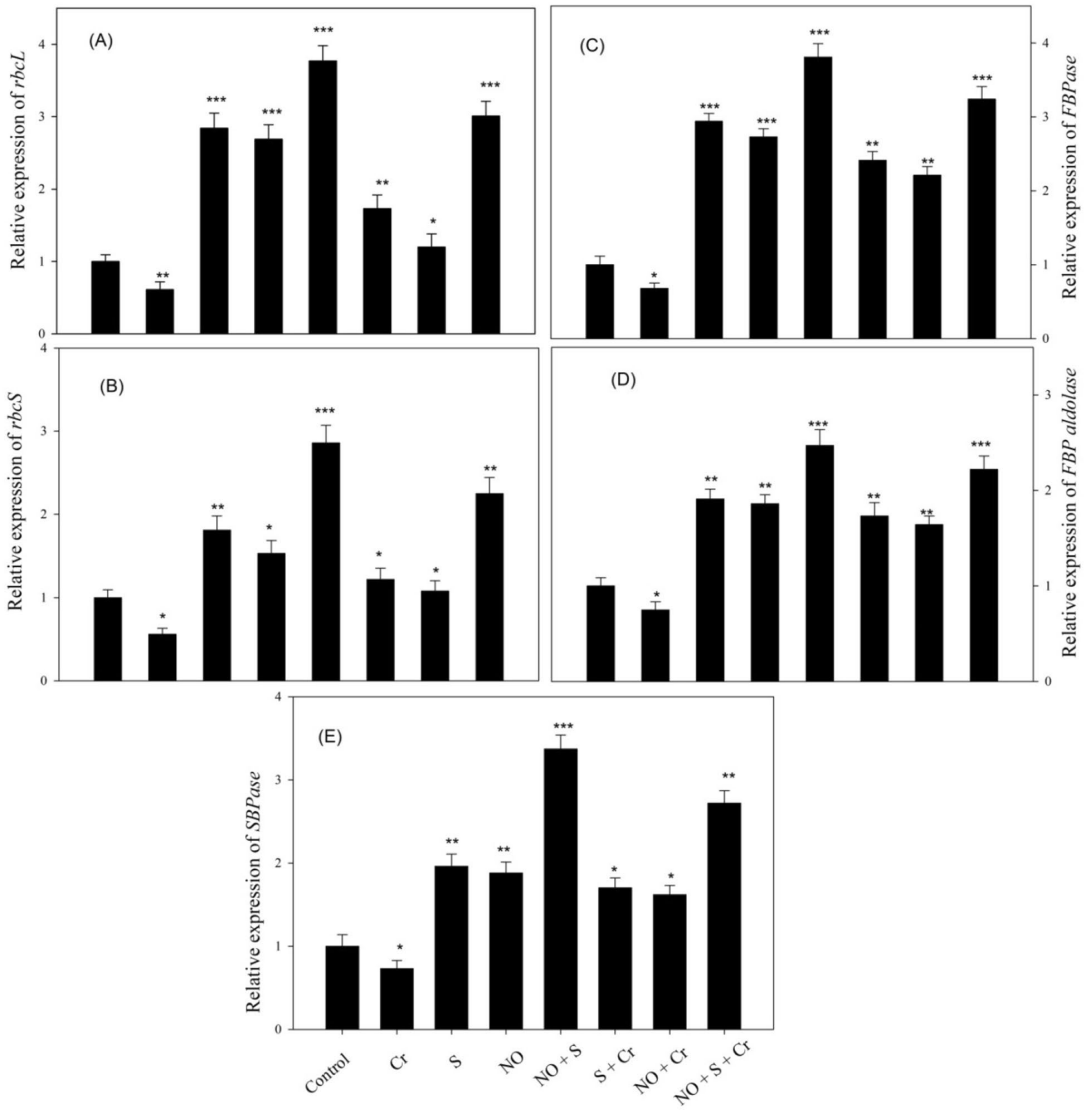
Relative expression of genes *rbcL* (**A**), *rbcS* (**B**), *FBPase* (**C**), *FBP* *aldolase* (**D**), and *SBPase* (**E**) in wheat (*Triticum* *aestivum* L.) leaves at 30 DAS. Plants were supplemented with 50 µM NO and/or treated with S (1.0 mM SO_4_^2−^) in the presence and absence of 100 µM Cr. Results are represented in relation to the respective control (control,1). The data is displayed as mean ± SE (*n* = 4). As established by Tukey’s posthoc multiple comparison test, values that differ significantly between the control and treatments are denoted with an asterisk (*p < 0.05, **p < 0.01, ***p < 0.001). *Cr* chromium, *DAS* d after sowing, *FBPase* fructose-1,6-bisphosphatase, *FBP* *aldolase* fructose-1,6-bisphosphatase aldolase, *NO* nitric oxide, *rbcL* ribulose-1,5-bisphosphate carboxylase/oxygenase large subunit, *rbcS* ribulose-1,5-bisphosphate carboxylase/oxygenase small subunit, *S* sulfur, *SBPase* sedoheptulose-1,7-bisphosphatase, *S* sulfur.

The relative transcript levels of genes involved in the Calvin cycle were examined. As shown in Fig. 3, the transcriptional level of these genes Rubisco large subunit (*rbcL*), Rubisco small subunit (*rbcS*), *FBPase*, *FBP* *aldolase,* and *SBPase* varied significantly with NO or S in comparison to the control or Cr-treated plants. However, the combined supply of NO and S significantly increased the transcriptional level of the genes, which were reduced by Cr stress.

However, in comparison to the control, the soluble sugar content was reduced by 25.2%, starch content by 23.3%, and total non-structural carbohydrate (NSC) content by 24.3% under Cr stress (Table 3). Contrarily, when NO or S was applied under no stress, the aforementioned parameters enhanced in comparison to the control. Moreover, S increased the soluble sugars, starch, and total NSC content by 23.2%, 23.7%, and 23.4%, respectively. Moreover, NO increased the soluble sugars, starch, and total NSC content by 20.9%, 20.7%, and 20.8%, respectively, under no stress, in comparison to the control. Equally, our results suggested that plants receiving both NO and S under no stress exhibited significantly higher soluble sugars, starch, and total NSC content by 40.3%, 36.0%, 38.4%, and 87.7%, 77.3%, and 83.1%, respectively, in comparison to the control and Cr-treated plants, respectively. With the application of S and NO in Cr-treated wheat plants, we observed a maximal increase in soluble sugars, starch, and total NSC content by 33.4%, 29.2%, 31.6%, and 78.5%, 68.5%, and 74.0% in comparison to the control and Cr stress plants, respectively.

### The involvement of GSH in NO and S-mediated alleviation of Cr stress and protection of photosynthesis

**Figure 4:**
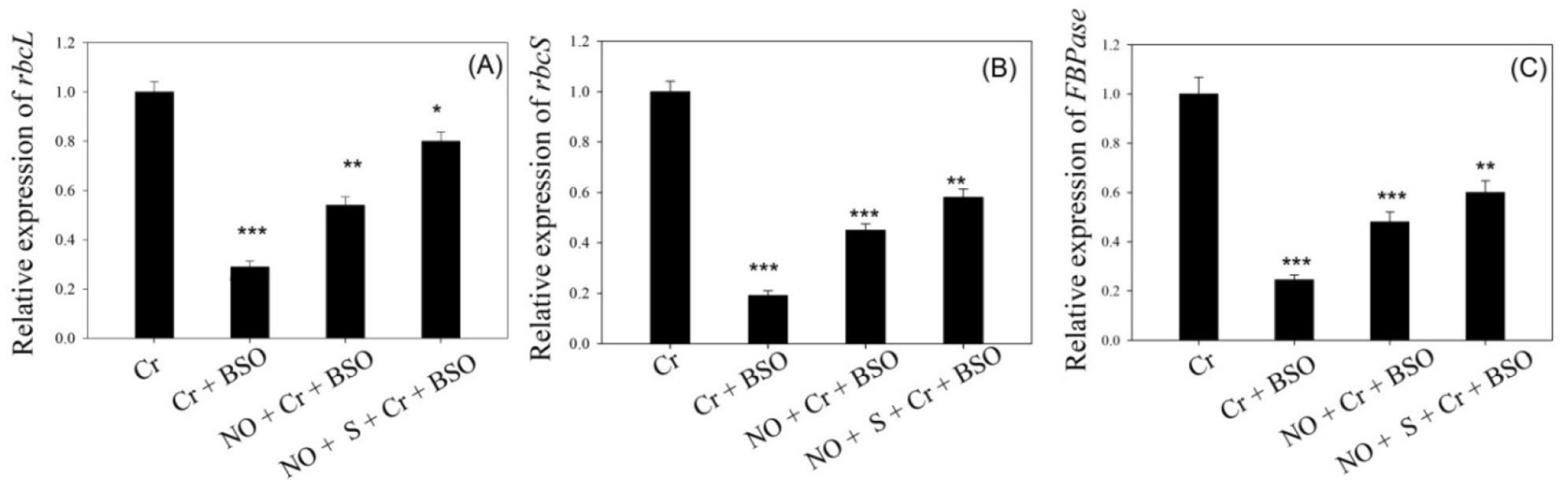
Relative expression of the genes *rbcL* (**A**), *rbcS* (**B**), and *FBPase* (**C**) in wheat (*Triticum* *aestivum* L.) leaves at 30 DAS. Plants were supplemented with 100 µM Cr, 100 µM Cr + 0.5 mM BSO, 100 µM Cr + 0.5 mM BSO + 50 µM NO and 100 µM Cr + 0.5 mM BSO + 50 µM NO + 1.0 mM SO_4_^2−^. Results are represented in relation to the respective control (control, 1). The data is displayed as mean ± SE (*n* = 4). As established by Tukey’s posthoc multiple comparison test, values that differ significantly between the control and treatments are denoted with an asterisk (*p < 0.05, **p < 0.01, ***p < 0.001). *Cr* chromium, *DAS* d after sowing, *FBPase* fructose-1,6-bisphosphatase, *NO* nitric oxide, *rbcL* ribulose-1,5-bisphosphate carboxylase/oxygenase large subunit, *rbcS* ribulose-1,5-bisphosphate carboxylase/oxygenase small subunit, *S* sulfur.

Application of NO with S increased GSH synthesis via S-assimilation, which reduced the negative effects of Cr on photosynthetic parameters. These observations were made when plants treated with Cr or Cr + buthionine sulfoximine (BSO) were compared to plants receiving BSO in the presence of NO and Cr or NO, Cr, and S (Table 4; Fig. 4). In comparison to plants treated with Cr, supplementation of BSO to plants treated with NO under Cr stress or plants treated with NO and S in the presence of Cr stress had more detrimental effects on *Pn*, maximum PS II efficiency, plant dry weight, and the activity and expression of Rubisco and FBPase enzymes. Moreover, under these treatments, the content of H_2_O_2_ was more, and, the content of GSH and activity of ATP-S were lowered compared with the Cr-treated plants. The maximal decrease in plant dry weight, *Pn*, maximal efficiency of PS II, and ATP-S activity and GSH content with the greatest H_2_O_2_ was observed in Cr plus BSO-treated plants compared to the other treatments. These plants showed decreased transcriptions for genes *rbcL,* *rbcS,* and *FBPase*. However, plants receiving BSO with the combined application of NO and S in the presence of Cr stress observed a lesser decrease in the above-mentioned parameters in comparison with the plants treated with NO plus BSO under Cr stress.

**Table 4:** Net photosynthesis, maximal efficiency of PS II, plant dry weight, the activity of Rubisco, FBPase, ATP-S, and the content of GSH and starch in wheat (*Triticum* *aestivum* L.) leaves at 30 DAS.

Treatments	Cr	Cr + BSO	NO + Cr + BSO	NO + S + Cr + BSO
H_2_O_2_ content (nmol g^−1^ dry weight)	30.9 ± 0.980	56.90 ± 0.990***	45.6 ± 0.980**	39.60 ± 0.830*
ATP-S activity (µmol g^−1^ protein s^−1^)	01.59 ± 0.041	01.27 ± 0.033**	01.39 ± 0.041*	01.51 ± 0.046 ns
GSH content (nmol g^−1^ dry weight)	072.0 ± 2.810	41.00 ± 1.620***	53.10 ± 1.910**	61.00 ± 2.010*
Net photosynthesis (µmol CO_2_ m^−2^ s^−1^)	07.10 ± 0.550	04.35 ± 0.240***	05.53 ± 0.440*	06.30 ± 0.510*
Maximal efficiency of PS II (Fv/Fm)	0.699 ± 0.002	0.605 ± 0.002**	0.660 ± 0.002*	0.685 ± 0.002 ns
Plant dry weight (g plant^−1^)	0.710 ± 0.057	0.620 ± 0.053***	0.660 ± 0.054*	0.690 ± 0.054 ns
Rubisco activity (µmol CO_2_ mg^−1^ protein min^−1^)	0.460 ± 0.012	0.230 ± 0.006***	0.290 ± 0.009**	0.380 ± 0.011*
FBPase activity (µmol NADPH mg^−1^ protein min^−1^)	0.550 ± 0.018	0.320 ± 0.016***	0.410 ± 0.017**	0.490 ± 0.017*
Starch content (mg g^−1^ dry weight)	18.5 ± 1.162	14.8 ± 1.010**	16.7 ± 1.150*	17.1 ± 1.158 ns

Plants were supplemented with 100 µM Cr, 100 µM Cr + 0.5 mM BSO, 100 µM Cr + 0.5 mM BSO + 50 µM NO and 100 µM Cr + 0.5 mM BSO + 50 µM NO + 1.0 mM SO_4_^2−^. The data is displayed as mean ± SE (*n* = 4). As established by Tukey’s posthoc multiple comparison test, values that differ significantly between the control and treatments are denoted with an asterisk (*p < 0.05, **p < 0.01, ***p < 0.001). *ATP-S* ATP-sulfurylase, *Cr* chromium, *DAS* d after sowing, *FBPase* fructose-1,6-bisphosphatase, *GSH* reduced glutathione, *NO* nitric oxide, *ns* non-significant, *S* sulfur.

## Discussion

One of the most commonly encountered heavy metal stress that limits plant growth and productivity is Cr stress. Wheat is an important staple crop that shows more sensitivity to high Cr stress concentrations^28^. In this regard, the role of S in enhancing NO efficacy in regulating responses to Cr stress has been investigated. The current work identified a sustainable approach in the reversal of Cr-inhibited activity of Calvin cycle enzymes, photosynthetic activity, and carbohydrate metabolism through the supplementation of NO and S.

As a result of excessive ROS production, oxidative stress develops in plants causes disruption of the cellular metabolism^6,9,28^, and in response to such conditions, plants activate the activity of the antioxidant system to restrict the oxidative stress. The present research highlighted that the accumulation of H_2_O_2_ and TBARS was limited when plants received NO and/or S. Studies have reported that NO and/or S supplementation decreased oxidative stress by enhancing the activity of the enzymatic antioxidants^21,29^. The enzymatic antioxidants APX and CAT catalyze H_2_O_2_ to H_2_O by dismutation and primarily transform H_2_O_2_ into H_2_O and O_2_, respectively^21,30^. On a similar line Singh et al.^22^ reported that the supplementation of NO, Ca, and S reversed the Cr-induced ROS accumulation and their associated damage in lipids and membranes by improving the antioxidant potential in tomato and brinjal. In our study, the exogenous NO markedly improved the S-assimilation with S treatment by increasing the activities of ATP-S and OASTL, and this crosstalk between NO and S contributes to the improved tolerance to Cr stress. These results are in line with research on NO, which showed considerable enhancement in the accumulation of Cys with the increased OASTL activity, lowering ROS and malondialdehyde levels in tomato seedlings under Cr stress^22,23^. Studies have reported that increased S enhanced tolerance to Cr stress via the stimulation of S-assimilation and its related enzymes^12,31^. Here, reduced GSH serves as a vital non-enzymatic antioxidant that played an important role in reducing Cr toxicity in wheat plants. Exogenous NO maintained a high GSH/GSSG ratio, indicating better protection and adaptation of plants under salt stress^20^. Khan et al.^32^ suggested that the continuous supply of Cys was necessary for maintaining high GSH levels in the GSH pool and redox homeostasis that impart tolerance to osmotic stress. A high concentration of reduced GSH interacts with NO and leads to the formation of GSNO, which serves as a NO pool in plants under stressful conditions^33^. The GSSG is reduced back to GSH by the nicotinamide adenine dinucleotide (NADPH)-dependent mechanism that is mediated by GR, and the GSNO is produced by the GSNOR-producing GSSG and ammonia. Thus, the rapid recycling of GSH is correlated with NO in continuing the pool of GSH and the redox state. The increased production of GSH is correlated with increased S-assimilation, and both were upregulated under oxidative stress conditions^21^. In the present study, this was due to the increased S supply that helped to protect wheat plants from Cr toxicity by GSH synthesis. When the GSH was present in high concentrations, it acted as a buffering system besides redox imbalance. It helped in plant growth and development under stress states by scavenging ROS accumulation in tomato plants^34^. Moreover, NO-induced GSH synthesis through the upregulation of genes encoding γ-ECS (gamma-glutamylcysteine synthetase) and glutathione synthetase. The induced GSH synthesis played an important role in detoxifying cadmium (Cd) and Cr stress in barrel medick and tomato seedlings, respectively^23,33^. Remarkably, our results confirm that the increased synthesis of GSH through the combined effect of NO and S provides potency to fight against Cr toxicity by enhancing the S-assimilation pathway. This is the first report on the response of plants to the application of NO with S under Cr stress in wheat.

The increased antioxidant activity with the application of NO with S also protected Chl disintegration and photosynthetic capacity under Cr stress. Interestingly, the availability of nutrients has been connected to the response of NO. Singh et al.^22^ reported that NO enhanced the uptake of nutrients necessary for enhancing Chl synthesis and photosynthesis. The supplementation of NO increased the uptake of S through enhanced S-assimilation and resulted in improved GSH synthesis in the present study. The increased GSH production by NO with S delivered an important regulatory loop under Cr stress that increased photosynthesis by increasing stomatal conductance. Besides, the Cr-treated plants influenced electron transport due to the destruction of antenna pigments and minimized the PS II efficiency and yield^35^. The reduced CO_2_ assimilation during Cr stress is caused by the decline in PS II efficiency and qP. In this regard, we found that NO and/or S-treated plants exhibited improved Chl content, photochemistry of PS II via reduced Cr effects. These results are relevant to the previous findings where photosynthetic characteristics and morphological characteristics improved with the application of NO and S in brinjal and tomato seedlings^22,23^. The published research has shown the NO and S-mediated mitigation of different abiotic stress in plants and enhancement in the photosynthetic efficiency by the influence on nitrogen (N) and S uptake^21,22,29^. But, to our knowledge, the interactive effect of NO with S in alleviating the Cr stress with increased photosynthetic and growth characteristics in wheat plants is unknown.

Photosynthesis is an important process that determines the carbohydrate synthesis required for the sustenance of plant growth and development under optimal and stressful conditions. Studies have indicated that Cr stress adversely affects photosynthesis through its influence on the Calvin cycle enzymes. It disrupts the electron transport and thylakoid membrane and inhibits carbon fixation by targeting the Calvin cycle enzymes^36^. SBPase and FBPase, two of these enzymes, play significant roles and are controlled by the redox potential through the ferredoxin/thioredoxin system^37^. Both SBPase and FBPase catalyze the irreversible reaction in the Calvin cycle, and FBPase controls the cycle's regeneration phase, and starch biosynthesis, and functions as a branch point intermediate^38^. d-Fructose-1,6-biphosphate is converted by the enzyme FBP aldolase into d-glyceraldehyde-3-phosphate and dihydroxyacetone phosphate, which are important steps of photosynthesis. Yildiz and Teri^39^ have reported that Cr stress downregulates photosynthesis through its effect on FBP aldolase and glutamine synthetase enzymes. This decrease was restored by Cys. Probably, S could have played a role in this increase of FBP aldolase as Cys is the first stable product of S-assimilation. Since Cys is related to S-assimilation, it has a significant impact on regulating Cr stress by GSH synthesis. It reverses the negative effect of stress-induced inhibition of photosynthesis. Besides, in a study on Cd and copper (Cu) stressed plants, NO improved the contents and activity of Rubisco and Rubisco activase, which decreased under stress^40^. NO exhibits metabolic crosstalk with the GSH pool and imparts stress tolerance^21^. Thus, this suggests that the combined application of NO and S helps restore the activities of the Calvin cycle enzymes under Cr stress through the increased production of GSH via the supply of S.

As we mentioned earlier in this report, plants under Cr stress significantly reduced the activity of Rubisco. This reduction was the result of lower transcriptional levels of genes such as *rbcL* *and* *rbcS*. In our study, NO and S improved the expression of these enzymes, which was efficacious in reducing the negative effects of Cr on photosynthesis. The increased expression of the Calvin cycle enzymes increases photosynthesis under Cr stress is in line with the report of Miyagawa et al.^41^. They observed that the overexpression of *FBP/SBPase* in plants resulted in higher sucrose, hexose, along with starch than in wild-type plants. Both two subunits *rbcL* and *rbcS* regulate the structure and/or function of Rubisco^42^. Tamoi et al.^43^ suggested that even a minor increase in the activity of FBPase contributed to starch synthesis, while SBPase in the regeneration of ribulose-1,5-bisphosphate (RuBP) and the enhanced chloroplastic FBPase or SBPase induced an increase in RuBP and favorably regulated photosynthesis in transgenic plants. It was noted that Cr metal ions replaced Mg^2+^ in the active site of Rubisco, which in turn affected Chl concentration in *Salvinia* *natans* exposed to wastewater high in Cr content^44,45^. Thus, Cr reduces the Calvin cycle enzymes, leading to reduced photosynthetic potential, as is observed in the present study, where a decrease in FBPase, FBP aldolase, and Rubisco occurred together with a decrease in starch and soluble sugars content. Iqbal et al.^46^ suggested that crosstalk of melatonin and H_2_S reduced oxidative stress by increasing antioxidant enzymes in wheat and increasing carbohydrate metabolism by increasing the soluble sugars and starch content in heat-stressed plants. Thus, the contents of soluble sugars are used here for defense against stress responses because they play a key role in numerous metabolic processes and act as a signal molecule to control various photosynthesis-related gene expressions. In another study, Ye et al.^47^ suggested that by promoting the activity of Rubisco, photosynthetic electron transfer, and Chl production, H_2_S enhanced photosynthesis. This shows that due to its signaling features, H_2_S is an essential mode of S metabolism and has greater particular relevance. In fact, S regulates a number of physiological mechanisms in plants^48^, as well as the photosynthetic apparatus and electron transport system, in addition to acting as coenzymes and prosthetic groups for ferredoxin necessary for N assimilation^49^. In this research, efforts were undertaken to explore the potentiality of combined NO and S in alleviating Cr-induced toxicity on carbohydrate metabolism. It is noteworthy that, in the present study, we found that NO and/or S supplementation not only maintained the antioxidant potential but also regulated the expression of genes related to the Calvin cycle and reduced the effects of Cr stress on wheat plants. The effects of NO and S on gene expression of the Calvin cycle enzyme and Cr stress mitigation in wheat plants have not been reported earlier.

**Figure 5:**
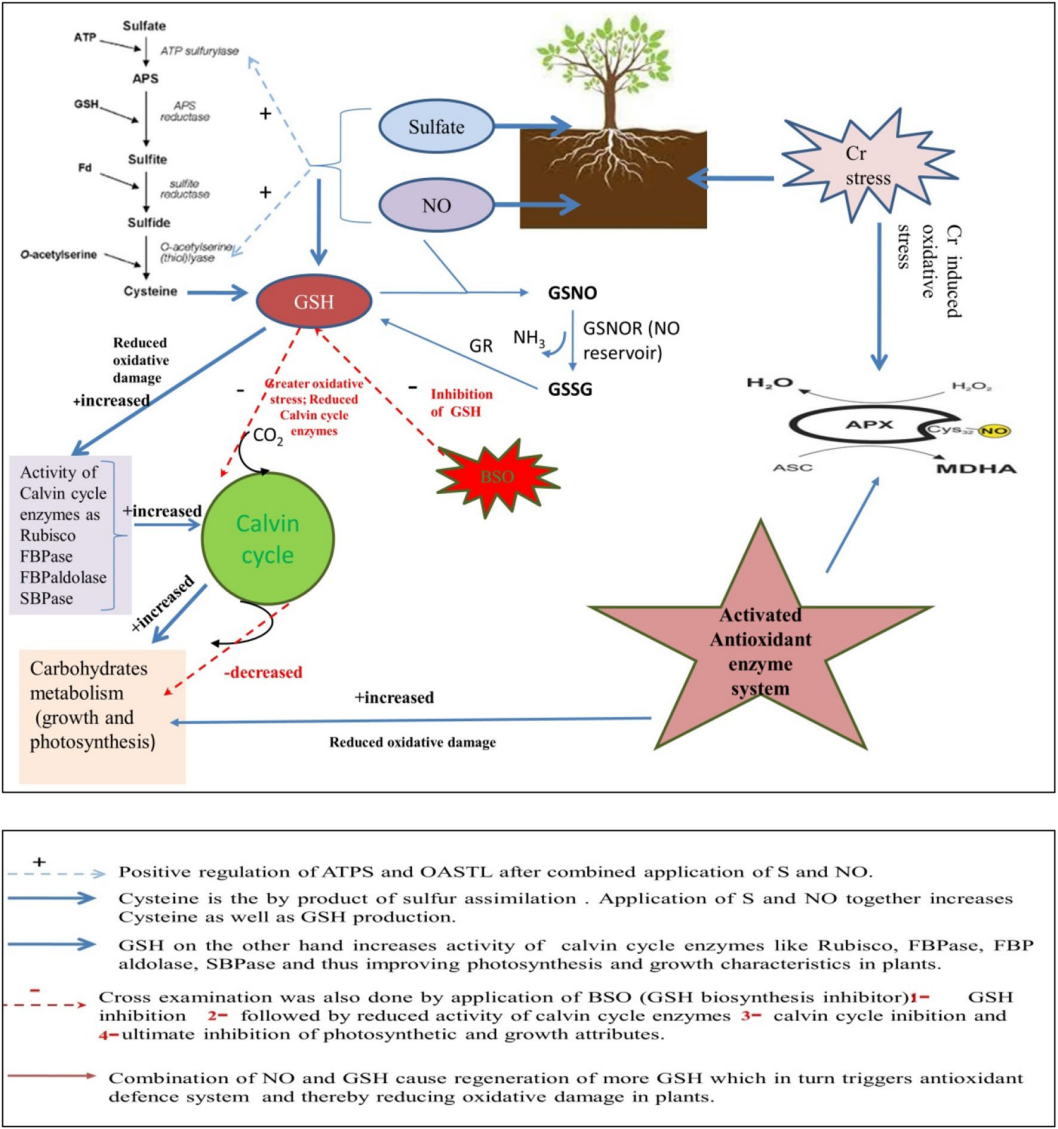
The combined effect of NO and S in the alleviation of Cr stress through the involvement of GSH and protection of photosynthesis.

The present study suggested that the enhanced GSH resulted from improved S-assimilation on NO and S supplementation was involved in photosynthetic protection under Cr stress. However, treatment of NO and S supplemented plants with BSO (GSH synthesis inhibitor) under Cr stress reduced GSH production and reversed the effects of NO and S on photosynthesis and the expression of the Calvin cycle enzymes. Therefore, the more conspicuous alleviation of Cr stress with NO with S and protection of photosynthesis involved GSH. Figure 5 shows the involvement of GSH in NO and S-mediated alleviation of Cr stress and the protection of photosynthesis.

The use of NO with S availability resulted in ROS scavenging and enhanced plant growth and photosynthesis and mitigation of Cr toxicity. The gene expression analysis of Rubisco subunits strongly suggests that NO and S treatment will positively regulate photosynthetic machinery in plants under Cr toxicity. The study also reveals the integration of physiological and biochemical processes with a special emphasis on the Calvin cycle enzymes and GSH production for Cr stress mitigation. This study could provide opportunities to exploit the mitigation of Cr stress with the combined effect of NO and S and develop Cr stress resilience in wheat crops. Future research should be focused on analyses of different photosynthesis and antioxidant enzymes genes under the influence of NO and S to have a more collaborative knowledge on Cr stress mitigation in plants. The research reported here has substantial application on the agricultural environmental system. The accumulation and adverse impact of high Cr concentration due to the anthropogenic activities may be reduced with the modified agricultural technology where supplementation of NO and S may be adopted as a part of the farming practice.

## Materials and methods

All of the plant material and experimental design, research, and field studies in the current research have fully complied with relevant institutional, national, and international guidelines and legislation. Wheat (*Triticum* *aestivum* L. cv. HD 2851) seeds were obtained from National Seeds Corporation, New Delhi, India. Seeds were surface sterilized with dilute mercuric chloride (HgCl_2_) solution followed by repetitive washing with distilled water. Further, seeds were sown in earthen pots with 23 cm diameter filled with sterilized acid-washed fine sand with a pH of 7.0 and particle size of about 125–250 µm. Sand purification was done beforehand with 17% (*w/v*) HCl (hydrochloric acid) for 6–7 h^50^. Transferred seeds were supplied with Hogland solution (300 mL) as a nutrient medium in the sand every alternate day. Soon after proper germination, 4 plants were maintained in each pot. Plants were allowed to grow in average day/night temperatures of 25/17 ± 3 °C, photosynthetically active radiation (PAR) at 650 µmol m^−2^ s^−1^, and 65 ± 5% relative humidity (RH) in the naturally lightened greenhouse of the Department of Botany, Aligarh Muslim University, Aligarh, India.

After 10 DAS, 100 µM Cr or 1.0 mM SO_4_^2−^ treatments were given in the form of potassium dichromate (K_2_Cr_2_O_7_) and MgSO_4_, respectively, along with the nutrient solution. For the equivalence of Mg in the treatment and control set, MgCl_2_ was used as described earlier by Nazar et al.^51^. Moreover, the foliar application of 50 µM sodium nitroprusside (SNP; NO donor) was sprayed on plants treated with or without 1.0 mM SO_4_^2−^ and 100 µM Cr at 20 DAS. The efficiency of NO on S-assimilation, GSH production, CO_2_ assimilation, Chl fluorescence quenching, the Calvin cycle enzymes activity, gene expression, carbohydrate accumulation, and reduction of Cr stress was determined in the presence of SO_4_^2−^ and Cr. A GSH biosynthesis inhibitor, 0.5 mM BSO was added to Cr, NO + Cr, and NO + Cr + S treated plants in order to assure the NO-mediated role on GSH production through S-assimilation in Cr stress alleviation at 20 DAS and compared with Cr-treated plants alone. The concentration of Cr, SO_4_^2−^, SNP, and BSO was selected on the basis of earlier findings^52–55^. Complete randomized designs were selected for treatment. Sampling was done at 30 DAS with 4 replicates (*n* = 4) for each treatment.

### Determination of oxidative stress

Protocols of Okuda et al.^56^ and Dhindsa et al.^57^ were followed to determine H_2_O_2_ and lipid peroxidation (TBARS) with slight changes. For the determination of H_2_O_2_ content_,_ leaf tissues (500 mg) were grounded in 2 mL of ice-cold 200 mM perchloric acid (HClO_4_) of the supernatant with 4 M potassium hydroxide (KOH). Further, centrifugation was done at 500×*g* for 3 min for the elimination of insoluble potassium chlorate (KClO_4_). The reaction mixture contained 1 mL of eluate, 400 µL of 12.5 mM 3-(dimethylamino)-benzoic acid in 0.375 M phosphate buffer having pH 6.5, 80 µL of 3-methyl-2-benzothialine hydrazone and 20 µL of peroxidase (0.25 units) in a final volume of 1.5 mL. Moreover, the reaction was started by the addition of peroxidase (at 25 °C), and at 590 nm the increase of absorbance was recorded.

For the determination of lipid peroxidation, the content of TBARS was expressed. Leaf tissues (500 mg) were crushed in 5 mL of 0.25% 2-thiobarbituric acid in 10% trichloroacetic acid, and the resulting mixture was heated to 95 °C for 30 min and quickly cooled in an ice bath. Then, centrifugation was done at 10,000×*g* for 10 min. 1 mL of the supernatant was mixed with 4 mL of 20% trichloroacetic acid which also contained 5% thiobarbituric acid. At 532 nm, the color's intensity was measure and subtracted the absorbance of the same at 600 nm to correct for nonspecific turbidity. Using the extinction coefficient (155 mM^−1^ cm^−1^), the content of TBARS was determined. The procedure is also published in our earlier findings by Fatma et al.^30^.

### Antioxidant system

Leaf tissues (200 mg) were homogenized with an extraction buffer comprising 0.05% (v/v) Triton X-100 and 1% (w/v) polyvinylpyrrolidone in 100 mM potassium-phosphate buffer in a cooled mortar and pestle (pH 7.0). The homogenate was centrifuged for 20 min at 15,000×*g* at 4 °C. After centrifugation, the supernatant was utilized for the enzymes assay. And, for the estimation of the activity of the APX enzyme, the extraction buffer was supplemented with 2 mM ascorbate.

Following Aebi^58^ method, CAT activity was determined at 240 nm and as an extinction coefficient at 0.036 mM^−1^ cm^−1^. In CAT activity, we monitored the rate of disappearance of H_2_O_2_ at 240 nm. The amount of enzyme required to decompose 1 µmol of H_2_O_2_ per minute at 25 °C is one unit.

However, for the assay of APX estimation, Nakano and Asada^59^ method were used with slight modifications. The assay combination included enzyme extract, phosphate buffer (50 mM, pH 7.0), 0.1 mM EDTA, 0.5 mM ascorbate, and 0.1 mM H_2_O_2_. A decrease in absorbance was recorded at 290 nm and the extinction coefficient was taken as 2.8 mM^−1^ cm^−1^. The amount of enzyme required to decompose 1 µmol of substrate per minute at 25 °C is one unit.

For estimating the activity of GR, we adopted Foyer and Halliwell^60^ method with certain modifications. The enzyme extract, phosphate buffer (25 mM, pH 7.8), 0.5 mM GSSG, and 0.2 mM NADPH were all included in the assay mixture. Here, we determined glutathione oxidation of NADPH at 340 nm and 6.2 mM^−1^ cm^−1^ as an extinction coefficient. The amount of enzyme required to decompose 1 µmol of NADPH per minute at 25 °C is one unit.

Griffith^61^ protocol was adopted for reduced glutathione assay. The chemical reaction involves sequential oxidation and reduction by 5,5′-dithiobis-2-nitro benzoic acid (DTNB) and lowering by NADPH, respectively in the presence of GR. For the specific GSSG assay, derivatization with 2-vinyl pyridine masks GSH. Using a mortar and pestle, leaf tissue (500 mg) was pulverized in liquid nitrogen and then suspended in 2 mL of 5% (w/v) sulfosalicylic acid before being centrifuged at 12,000×*g* for 10 min. A 300 µL aliquot of the supernatant was taken out, and 18 µL of 7.5 M triethanolamine was added to neutralize it. The concentrations of GSH and GSSG were then determined using a 150 µL sample. To mask the GSH by derivatization and enable the subsequent detection of GSSG alone, another sample was pretreated with 3 µL of 2-vinylpyridine for 60 min at 20 °C. 50 µL portions of each of the two sample types were combined with 700 µL of 0.3 mM NADPH, 100 µL of DTNB, and 150 µL of a buffer containing 125 mM sodium phosphate and 6.3 mM EDTA (pH 6.5). The addition of a 10 µL aliquot of GR (5U mL^−1^) was followed by monitoring the change in absorbance at 412 nm at 30 °C. GSH was used to prepare a standard curve with a range of 5–55 nmol. A standard curve with a range of 1–5 nmol was utilized for GSSG. The ratio of GSH to GSSG was used to determine the redox state.

### S-assimilation enzyme assay and Cys content

Estimation of ATP-S and OASTL activity was conducted using Lappartient and Touraine^62^ and Riemenschneider et al.^63^ methods, respectively.

For the assay of ATP-S, leaf tissue (200 mg) was ground at 4 °C using a 1:4 (w/v) tissue to buffer ratio in a buffer containing 10 mM Na_2_EDTA, 20 mM Tris–HCl (pH 8.0), 2 mM dithiothreitol (DTT), and 0.01 g mL^−1^ PVP. After that, centrifuge the homogenate at 20,000×*g* for 10 min at 4 °C. Further, in vitro ATP-S assay was conducted using the supernatant. Utilizing the molybdate-dependent production of pyrophosphate, the enzyme activity was determined. The reaction was started by adding 0.1 mL of extract to 0.5 mL of the reaction mixture, which comprised 0.032 units mL^−1^ of sulfate-free inorganic pyrophosphate in an 80 mM Tris–HCl buffer, 7 mM MgCl_2_, 5 mM of Na_2_MoO_4_, and 2 mM of Na_2_ATP (pH 8.0) in an Eppendorf tube. The same reaction mixture received another aliquot from the same extract, but this time Na_2_MoO_4_ was not present. The amount of phosphate in the reactions was then measured using a spectrophotometer after 15 min of incubation at 37 °C.

For the estimation of OASTL, extracts were prepared by using leaf tissue (200 mg) and 1 mL 20 mM Tris-HCl. For further homogenization, the mixture was centrifuged for 10 min at 13,000×*g*. Then, 5 mM OAS, 5 mM Na_2_S, 33.4 mM dithiotreitol, 100 mM Tris–HCl (pH 7.5), and 50 µL enzyme extract were all included for OASTL activity.

To determine the Cys content, Na_2_S was used to start the reaction, which was then incubated at 37 °C for 30 min before being stopped by the addition of 1 mL of acidic ninhydrin reagent (0.8% ninhydrin [w/v] in 1:4 concentrated HCl:HOAc)^64^. To allow colour development, the samples were heated at 100 °C for 10 min, then cooled on ice. After stabilizing the colour complex with 900 µL of 70% ethanol added to 100 µL of samples, the absorbance was recorded at 560 nm. The amount of Cys was determined using a calibration curve that was generated under similar conditions for standard Cys.

### Determination of growth and photosynthetic parameters

Leaf area was calculated with the leaf area meter (LA-211; Systronics, New Delhi, India). Plant fresh weight was recorded. Plants were maintained at 80 °C in an oven till constant weight for dry weight estimation. Photosynthetic parameters, *Pn*, *Ci*, and *Gs* were calculated by an Infrared Gas Analyzer (CID-340, Bio-Science, Washington, WI, USA). The sampling was done at PAR ~ 680 µmol m^−2^ s^−1^, temperature ~ 22 °C, and relative humidity ~ 70%.

SPAD value was recorded using SPAD chlorophyll meter (SPAD 502 DL PLUS, Spectrum Technologies, Plainfield, IL, USA) and the Junior PAM chlorophyll fluorometer (Heinz Walz GmbH, Eichenring, Effeltrich, Germany) was used for the measurement of Chl fluorescence. Before the fluorescence measurements, leaves (mainly from the top of the plants) were adapted to the dark for 20 min. With a light intensity of 131 µmol m^−2^ s^−1^ (low beam intensity), maximum (Fm) and minimal fluorescence (Fo) was studied in dark-adapted leaves. Maximum fluorescence (Fm') and minimal fluorescence (Fo') in comparable leaves in the light-adapted condition were calculated using saturating light intensity at 830 µmol m^−2^ s^−1^, which was well balanced with steady-state fluorescence (Fs). The variable fluorescence (Fv and Fv′) was calculated using the fluorescence values Fm–Fo and Fm′–Fo′. The PS II system's intrinsic efficiency, represented by Fv′/Fm′, and its actual efficiency, represented by Fm′–Fs/Fm′, were calculated. Additionally, using fluorescence characteristics determined in both light- and dark-adapted conditions, NPQ and qP were assessed.

### Measurements of carbohydrate content

Starch estimation was determined spectrophotometrically at 620 nm by the use of anthrone reagent and glucose as the standard^65^. Dried leaf tissues were grounded and filtered by using a 1 mm sieve. The powdered material about 100 mg was added to 5 mL of 80% ethanol in a 10 mL centrifuge tube. The mixture was first incubated for 30 min at 80 °C in a water bath shaker before being centrifuged for 5 min at 4000×*g*. After that, 80% ethanol was used to extract the pellets. An evaporation process was done for the removal of the ethanol. The starch in the residue was liberated after 15 min in a boiling bath with 2 mL of distilled water and cooled to room temperature. After that, 9.2 mol l^−1^ HClO_4_ (2 mL) was used to hydrolyze leaf starch for 15 min. The samples were then centrifuged at 4000×*g* for 10 min after being given distilled water (4 mL). A second extraction of the residue was performed using 4.6 mol L^−1^ HClO_4_ (2 mL). Until 25 mL, the supernatants were collected, blended, and mixed with distilled water.

Xu et al.^66^ protocol was used for soluble sugars. For each treatment, top leaves that had fully expanded were collected in order to estimate the soluble sugar content. After drying in an oven at 80 °C, leaf samples were ground into a fine powder. Using 10 mL of 80% ethanol, the dried sample (100 mg) was extracted, and it was then heated in a water bath to 80–85 °C for 30 min. After centrifugation of the extract, the supernatant was transferred to a 100 mL volumetric flask. This process was done three times. At 80–85 °C, the alcohol extract was evaporated in a water bath. The flask was filled with a mixture of all three supernatants, and 100 mL of distilled water was then added. Anthrone reagent was used to determine the amount of soluble sugars in an extract, and a spectrophotometer was used to measure the reaction mixture's absorbance at 630 nm. Soluble sugars and starch content are expressed as mg g^−1^ dry weight.

The NSC content was the sum of the soluble sugar content and starch content (mg g^−1^ dry weight).

### Measurement of the Calvin cycle enzymes activity

Rubisco activity was determined according to Usuda^67^ method. The conversion of 3-phosphoglycerate to glycerol 3-phosphate occurs at 30˚C and 340 nm with the addition of enzyme extract. Leaf tissue was homogenized in an ice-cold extraction buffer comprising 0.25 M Tris–HCl (pH 7.8), 0.05 M MgCl_2_, 0.0025 M EDTA, and 37.5 mg DTT using a chilled mortar and pestle. Centrifuging the homogenate at 10,000×*g* for 10 min at 4 °C. A test for the enzyme was conducted using the resultant supernatant. The reaction mixture included 1 U each of glyceraldehyde 3-phosphodehydrogenase and 3-phosphoglycerate kinase, 100 mM Tris–HCl (pH 8.0), 40 mM NaHCO_3_, 10 mM MgCl_2_, 0.2 mM NADH, 4 mM ATP, 0.2 mM EDTA, 5 mM DTT, and 0.2 mM RuBP).

For FBPase and FBP aldolase, activity estimation Zhang et al.^68,69^ was followed. Leaf sample was homogenized in 4 mL of ice-pre-cooled buffer containing 50 mM MgCl_2_, 2 mM EDTA, 2% PVP, and 1%-mercaptoethanol. The homogenates were centrifuged at 15,000×*g* for 20 min at 4 °C and the supernatant was used to calculate the enzyme activity. The assay medium for FBP contains 2–4 units per mL of phosphoglucoisomerase and glucose-6-phosphate dehydrogenase in addition to 30 mM Hepes–KOH (pH 8.2), 5 mM MgCl_2_, 5 mM DTT, 0.5 mM NADP, and 5 mM FBP. FBP was added to the reaction to start it, and the rate was measured 10 to 15 min after the start of the assay. The activity of FBPase was expressed in (µmol NADPH mg^−1^ protein min^−1^). The mixture for the FBP aldolase assay contained 30 mM Hepes–KOH (pH 7.6), 10 mM FBP, 0.25 mM NADH, and 2–4 units per mL of each of alpha-glycerol-3-phosphate dehydrogenase and triose phosphate isomerase. The reaction was initiated by adding FBP. The activity of FBP aldolase was expressed in (µmol NADH mg^−1^ protein min^−1^).

Spectrophotometric estimation was performed for SBPase^70^. Leaf samples were thoroughly ground in liquid nitrogen and then transferred to 1 mL extraction buffer containing 50 mM Hepes (pH 8.2), 5 mM MgCl_2_, 1 mM EDTA, 1 mM EGTA, 10% glycerol, 2 mM benzamidine, 2 mM amino caproic acid, 0.5 mM PMSF, 10 mM DTT. After centrifuging, the supernatant was collected and run through a NAP-10 column that had been equilibrated with a desalting buffer. For the test, 80 mL of assay buffer (50 mM Tris; pH 8.2, 15 mM MgCl_2_, 1.5 mM EDTA, 10 mM DTT, and 2 mM SBP) was added to 20 mL of protein samples, which were then incubated at 25 °C for 5 min. The reaction was stopped by adding 50 µL of 1 M perchloric acid. The samples were then centrifuged for 5 min and the supernatant was assayed for phosphate. 850 µL molybdate solution (0.3% ammonium molybdate in 0.55 M H_2_SO_4_) was incubated with 50 µL samples and phosphate standards (0–0.5 mM NaH_2_PO_4_) for 10 min at room temperature. Following the addition of 150 µL of malachite green (0.035% malachite green, 0.35% polyvinyl alcohol), the samples were incubated for an additional 45 min at room temperature. Using a spectrophotometer, the reaction mixture's absorbance was recorded at 620 nm. The activity of SBPase was expressed in (µmol mg^−1^ protein s^−1^).

### Quantitative real time-PCR analysis

Freshly crushed leaves (~ 100 mg) were used for the analysis of gene expression by using the instruction kit. RNA extraction was followed by purification using Trizol reagent (Invitrogen, Carlsbad, CA, USA). 0.2 µg RNA was combined with cDNA using an oligo (dT) primer and M-MLV reverse transcriptase (Promega, Madison, WI, USA). Specific primers (as shown in Table S1) and SYBR Green as a fluorescent dye (Toyobo, Osaka, Japan) were used for the qRT-PCR. The *actin* (housekeeping gene) was used as an internal control. The relative amount of the target gene expression was determined by the 2^−ΔΔCT^ method.

### Statistical analysis

By using SPSS 17.0 software (SPSS Inc., Chicago, IL, USA) for Windows 10, data were statistically measured using analysis of variance (ANOVA) and Tukey's posthoc multiple comparisons test at *p* < 0.05 or *p* < 0.01. The data were represented as mean ± SE (*n* = *4*).

## Supplementary Information

Supplementary Information.

## Data Availability

Data supporting the findings of this work are provided in the paper and its Supplementary Information file. All other raw data that support this paper and other findings of this study are available from the corresponding author upon reasonable request. Data and materials will be shared with no restrictions on the availability of raw or processed data via a material transfer agreement.
