# Supplementary material for: Sulfur supplementation enhances nitric oxide efficacy in reversal of chromium-inhibited Calvin cycle enzymes, photosynthetic activity, and carbohydrate metabolism in wheat
Source: Sci Rep. 2023 Apr 26;13:6858. doi: 10.1038/s41598-023-33885-7 (PMC10133275; doi:10.1038/s41598-023-33885-7)
Supplement: Supplementary file 1 — Supplementary Information. [file 41598_2023_33885_MOESM1_ESM.doc]

Supplementary File S1.

**Sulfur supplementation enhances nitric oxide efficacy in reversal of chromium-inhibited Calvin cycle enzymes, photosynthetic activity, and carbohydrate metabolism in wheat**

Mehar Fatma^1^, Zebus Sehar^1^, Noushina Iqbal^2^, Ameena Fatima Alvi^1^, Gholamreza Abdi^3^, Nafees A. Khan^1,*^ and Charalampos Proestos^4,*^

^1^Department of Botany, Aligarh Muslim University, Aligarh 202002, India.

^2^Department of Botany, Jamia Hamdard, New Delhi 110062, India.

^3^Department of Biotechnology, Persian Gulf Research Institute, Persian Gulf University, Bushehr, 7516, Iran.

^4^Department of Chemistry, National and Kapodistrian University of Athens, Panepistimiopolis Zographou, 15771 Athens, Greece.

*Corresponding author: naf9.amu@gmail.com, [harpro@chem.uoa.gr](mailto:harpro@chem.uoa.gr)

These authors contributed equally to this work

**Table S1 :**Primer sequences and data used for RT-PCR analysis.

| **S.No.** | **Gene** | | | **Forward primer** | | **Reverse primer** |
| --- | --- | --- | --- | --- | --- | --- |
| 1 | *rbcL* | | | ACCGATGGGCTTACCAGTCT | | ATTCGCAAATCCTCCAGACG |
| 2 | *rbcS* | | | ATGGGTTCCCTGCGTTGA | | CCTGAGATGAGTCGGTGC |
| 3 | *FBPase* | | | GGGAGAGGACCAGAAAAA | | GGCTGTAGATGCCAAAGA |
| 4 | *FBP aldolase* | | | CTCGTGCTGCTGCTTACT | | CTGCCCAAACTTTCTGTG |
| 5 | *SBPase* | | | GCTGACAACCCAACCAA | | CATCCGACCATCACGAA |
| **Reference gene primer sequences used for quantitative RT-PCR** | | | | | | |
| 1 | | *Actin* | TGGACTCTGGTGATGGTGTTA | | CAATGAGGGATGGCTGGAAAA | |
